# Design of Cyclodextrin-Based Functional Systems for Biomedical Applications

**DOI:** 10.3389/fchem.2021.635507

**Published:** 2021-02-17

**Authors:** Wanjia Xu, Xiumei Li, Liang Wang, Siyuan Li, Shengnan Chu, Jiachun Wang, Yijia Li, Jinxing Hou, Quan Luo, Junqiu Liu

**Affiliations:** ^1^ State Key Laboratory of Supramolecular Structure and Materials, College of Chemistry, Jilin University, Changchun, China; ^2^ Key Laboratory of Emergency and Trauma, Ministry of Education, College of Emergency and Trauma, Hainan Medical University, Haikou, China; ^3^ Key Laboratory for Molecular Enzymology and Engineering of Ministry of Education, School of Life Sciences, Jilin University, Changchun, China

**Keywords:** cyclodextrin, selective recognition, inclusion complexes, functional materials, biomedical applications

## Abstract

Cyclodextrins (CDs) are a family of α-1,4-linked cyclic oligosaccharides that possess a hydrophobic cavity and a hydrophilic outer surface with abundant hydroxyl groups. This unique structural characteristic allows CDs to form inclusion complexes with various guest molecules and to functionalize with different substituents for the construction of novel sophisticated systems, ranging from derivatives to polymers, metal-organic frameworks, hydrogels, and other supramolecular assemblies. The excellent biocompatibility, selective recognition ability, and unique bioactive properties also make these CD-based functional systems especially attractive for biomedical applications. In this review, we highlight the characteristics and advantages of CDs as a starting point to design different functional materials and summarize the recent advances in the use of these materials for bioseparation, enzymatic catalysis, biochemical sensing, biomedical diagnosis and therapy.

## Introduction

**FIGURE 1:**
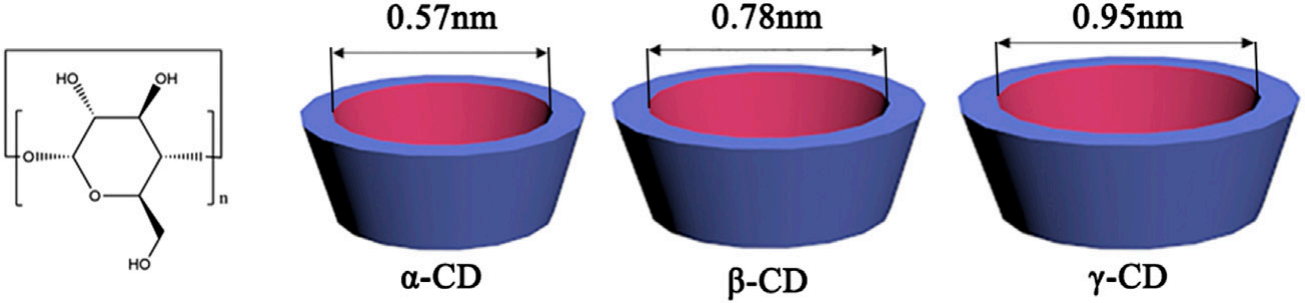
The structures of α-CD (n = 6), β-CD (n = 7), and γ-CD (n = 8).

CDs are well-known macrocyclic compounds that contain five or more α-D-glucopyranoside units linked by α-1,4-glycosidic bonds in the shape of a hollow truncated cone. In nature, α-, β-, and γ-CDs are three main types of members with six, seven, and eight units of glucose, respectively (Saenger, 1980) (see Figure 1). These molecules have a distinctive feature in their stereochemical structures, a relatively hydrophobic inner cavity with the diameter increased with the number of glucose units and a hydrophilic outer surface covered with abundant hydroxyl groups (Chen and Jiang, 2011). Thus, CDs can provide a favorable microenvironment to form inclusion complexes with various organic and bioactive molecules in different stoichiometry ratios (1:1, 1:2, 2:1 or 2:2) (Szejtli, 1998; Pinho et al., 2014). The selectivity of host-guest recognition arises from a size/shape-matching mechanism that requires the guest molecules to be trapped entirely or partially into the apolar CD cavities (Liu and Chen, 2006). The hydroxyl groups of CDs are also involved in the binding processes via electrostatic forces, Van der Waals, and hydrogen bonding interactions (Liu and Guo, 2002). The binding strength relies on the synergistic effect of these weak and reversible noncovalent interactions. This encapsulation ability will change the physiochemical properties of the included guest molecules, which has been widely explored for practical applications in biomedical fields by developing CD-based functional systems (Uekama et al., 1998; Davis and Brewster, 2004).

Natural CDs are hydrophilic, but exhibit a relatively low aqueous solubility due to the formation of aggregates via strong intermolecular hydrogen bonding (Coleman et al., 1992), which limits the range of their applicability. This shortcoming may cause nephrotoxicity when there is an accumulation of insoluble cyclodextrin crystals or cyclodextrin-cholesterol complexes in kidneys (Frijlink et al., 1991). Over the past few decades, a great effort has been devoted to improving the performances of CDs with the desired properties through various chemical modifications (Bellia et al., 2009; Řezanka, 2019). CDs have three types of hydroxyl groups, including the easily modified 6-OH groups on the upper rim and the most acidic 2-OH and the least accessible 3-OH groups on the lower rim. The different reactivity of these hydroxyl groups offers the possibility of regioselective substitution to yield more than 11,000 cyclodextrin derivatives by grafting with a large amount of functional groups (e.g., amines, amino acids, peptides, and aromatic groups) for improving their solubility and complexation capacity. In addition, the structural complexity can also be widened by the formation of inclusive complex between the bridged/branched CDs and guest molecules to construct more sophisticated supramolecular systems such as polymers, metal-organic frameworks, hydrogels, and other supramolecular assemblies (Nakahata et al., 2017; He et al., 2019; Zhang et al., 2020). These high-level superstructures allow for the functionalization with the bioactive moieties through supramolecular interactions or physical entrapment to achieve diverse functions by designing molecular receptors, delivery vectors, enzyme mimics, and fluorescence indicators.

In this review, the recent advance in the development of CD-based functional systems was summarized. The design strategies, physicochemical properties, and functional diversity of different CD derivatives and their high-level assemblies were discussed in detail. We also highlighted the unique advantages of these materials to achieve excellent performances, which show great potential in the field of bioseparation, enzymatic catalysis, biochemical sensing, biomedical diagnosis, and therapy for biomedical applications.

## Chiral Recognition for Bioseparation

Chiral discrimination is a hot topic in the field of biomedicines. This interest is driven by the different bioactivities and biotoxicities of chiral molecules when they participate in the biochemical processes through specific interactions with the biomolecules. Since the possibility of chiral separation was first demonstrated by using chiral stationary phases for chromatography in 1980 (Berthod, 2010), a wide variety of powerful techniques based on chromatography and electrophoresis have been developed (Sánchez-López et al., 2015). Chiral selectors act as a key factor to achieve enantioselective resolution by the formation of diastereomeric complexes with chiral analytes via intermolecular interactions, such as ion interactions, π-π interactions, van der Waals interactions, and hydrogen bonds. CDs are widely used chiral selectors, which have the following advantages (Rezanka et al., 2014): 1) The OH groups can be modified to design diverse derivatives for enantioseparation in different environments, including aqueous, polar organic, and nonpolar organic and 2) CDs can provide a chiral lipophilic cavity for selective recognition of nonpolar analytes without strict structural restriction.

### Drug Enantioseparation

**FIGURE 2:**
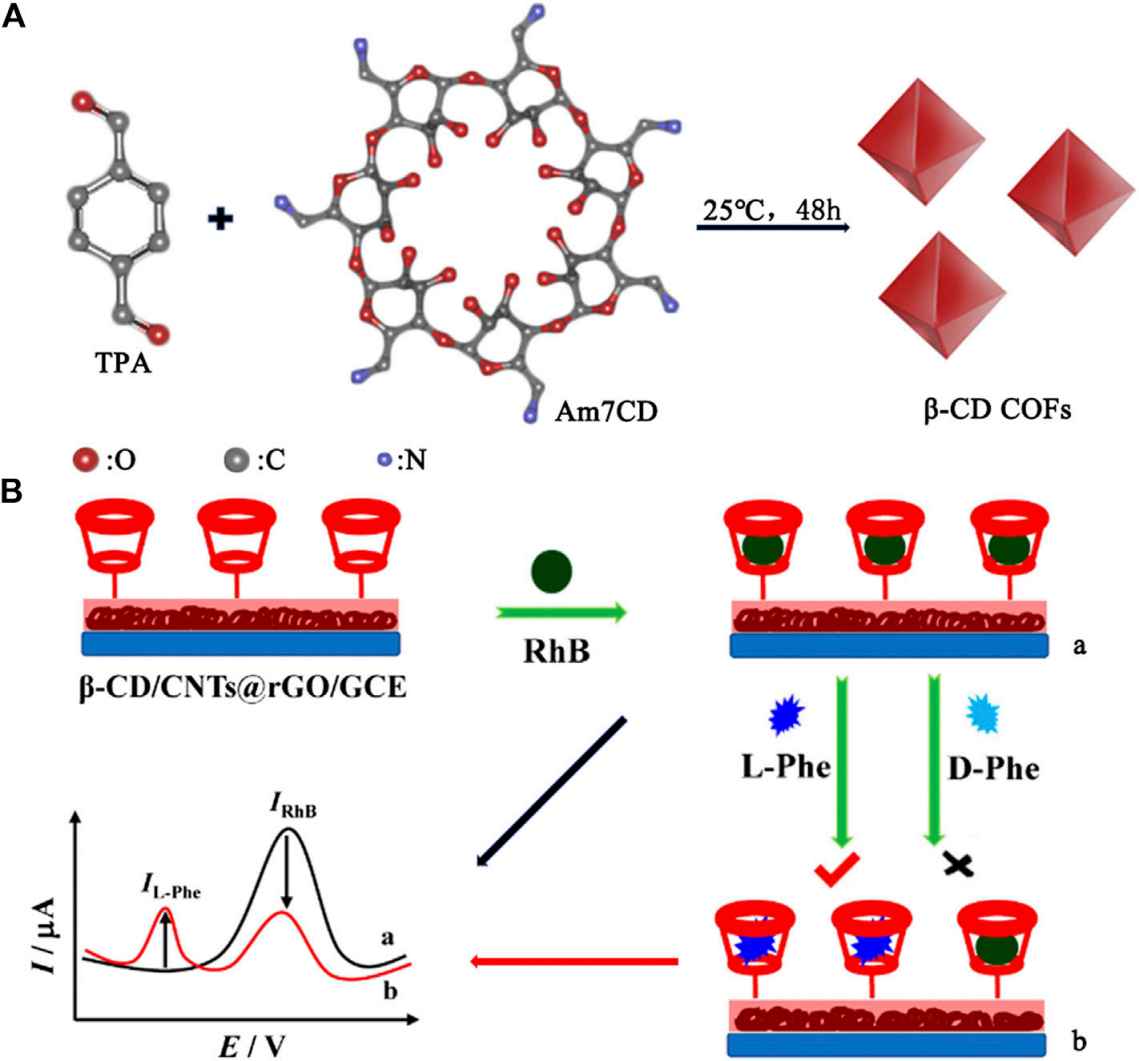
**(A)** The preparation of the chiral stationary phase: β-CD COFs. **(B)** The electrochemical sensor platform for recognition chiral phenylalanine enantiomers.

Chiral drugs account for more than 40% of the market share, which involves the treatment of a variety of diseases (Calcaterra and D’Acquarica, 2018). The use of pure drug stereoisomers will elicit more exact therapeutic effects to guarantee the safety. Since the United States Food and Drug Administration (FDA) and the European Committee for Proprietary Medicinal Products (CPMP) have claimed that the property of each enantiomer must be studied before it is marketed as one of the enantiomers or racemic drugs in 1992 (FDA, 1992), the demand for chiral drugs is met by developing the methods of chiral drug detection and separation. Native CDs exhibit enantiorecognition to differentiate enantiomeric species through the formation of diastereomeric complexes, which extend the applicability of current methods by adding to the stationary phase or the background electrolyte. Moreover, a large number of derivatives (e.g., neutral, cationic, and anionic CDs) were also created by introducing neutral or charged functional groups (Fillet et al., 2000; Zhou et al., 2015). These charged CDs have the obvious advantages of good solubility, increased cavity depth, and strong complexation ability with chiral drugs, which further improve the efficiency, time, and consumption of enantioseparation processes. For example, Yu and coworkers (Sun et al., 2019) simplified the preparation process by bonding a per-4-chlorophenylcarbamate-β-CD to chiral stationary phase. The resolution and selectivity of voriconazole reached 16.80 and 15.41. Chromatographic studies showed that 4-chlorophenylcarbamate group enhance the interactions of analyte–chiral substrate including hydrogen bonding, π–π and dipole–dipole interactions. In Figure 2A, Ji et al. used the condensation reaction of Heptakis (6-amino-6-deoxy)-β-CD (Am7CD) and terephthalaldehyde (TPA) to fabricate β-CD covalent organic frameworks (β-CD COFs) (Wang et al., 2019), a new chiral stationary phase that can achieve baseline separation of six chiral drugs (including (±)-sotalol, (±)-terbutaline, (±)-propranolol, (±)-metoprolol, (±)-salbutamol, and (±)-esmolol). The hydroxyl and amino groups in Am7CD provided additional driving forces to form inclusion complexes in addition to hydrogen bonds and van der Waals forces. And the porous material COF made CD units integrate, providing more interaction sites.

### Amino Acid Enantioseparation

Amino acids possess Levorotatory (L-)/dextrorotatory (D-) enantiomer pairs due to their asymmetric carbon center bonding to four different groups. These amino acid enantiomers have dissimilar biochemical function and properties: L-amino acids are the structural units of proteins found abundantly in organisms; however, D-amino acids seldom exist in higher animals and are often associated with various diseases such as Alzheimer (Sievers et al., 2011), schizophrenia (Chumakov et al., 2002), and amyotrophic lateral sclerosis (Paul and de Belleroche, 2012). Quantitative chiral separations and identification of some important D-amino acids like D-serine and D-aspartate are beneficial for studying the pathological changes (Szökő et al., 2016). Many experimental results revealed that cyclodextrins show a selectivity for L-amino acids, while the complexes of cyclodextrin and transition metal cations have specific interactions with D-amino acids to create sufficient mobility differences for effective separation (Chiu, 2013). In Figure 2B, a highly selective and sensitive electrochemical sensor for recognition chiral phenylalanine enantiomers (D-and L -Phe) was constructed based on CNTs@rGO (carbon nanotubes wrapped with reduced graphene oxide) allied with β-CD (Yi et al., 2019). In this model system, rhodamine B (RhB) was introduced as a probe and recognized by β-CD through host-guest interactions exhibiting remarkable oxidation peak current. In the presence of L-Phe, the peak current of RhB decreased and the peak current of L-Phe appeared due to the stronger interaction between L-Phe and β-CD. However, there was only a weaker interaction between β-CD and D-Phe. Nagy et al. used CDs in conjunction with a cationic group for high-resolution separation of D- and L-Ser, Asp and Thr in short times (Nagy et al., 2018). Using less ion manipulations and serpentine ultralong path with extended routing ion mobility (IM) platform, analyte ions are separated in extremely short times according to their mobilities and mass-to-charge ratio.

### Psychoactive Substance Enantioseparation

Psychoactive substances can act on the central nervous system to change the mood, consciousness, and behavior temporarily. Common psychoactive substances include derivatives, dissociative substances, opioids, and novel neuroactive substances. Many psychoactive substances possess a stereogenic center or contain positional isomers with the side chains on different substituent positions of a phenyl ring (e.g., ortho-, meta, or para-substitution). These enantiomers differ in their pharmacological effects, potency, or toxicity. Therefore, it is necessary to select appropriate chiral selectors for enantioseparation of psychoactive substances (Schmid and Hagele, 2020). CDs are one of the ideal candidates due to their multivalent weak interactions with psychoactive substances. The enantioseparation efficiency relies on the chiral recognition mechanism of CDs (Gübitz and Schmid, 2008), involving the inclusion of bulky hydrophobic groups into the chiral CD cavity and the second interactions between the hydroxyl groups at C2/C3 positions of CDs and the hydrophilic groups of psychoactive substances. Schmid and coworkers (Hägele et al., 2019) presented a chiral capillary zone electrophoresis method using four different β-CDs, namely, native β-CD, acetyl-β-CD, 2-hydroxypropyl-β-CD, and carboxymethyl-β-CD, as chiral selectors for the enantioseparation of 61 cathinone derivatives. Different modifications on their external surfaces had a huge impact on the formation and stability of inclusion compounds, so the separation of cathinone derivatives by the four β-CD derivatives was different. Under the optimized conditions, 58 of 61 tested cathinone derivatives were partially or baseline separated with at least one of the different CD-electrolytes within 40 min.

## Delivery Vectors for Biomedical Therapy

The development of drug delivery system is an effective strategy to improve the curative efficacy and safety of therapeutic molecules. Among them, CDs and their derivatives show the ability to increase the solubility, stability, dissolution rate, and bioavailability of poorly soluble drugs through the formation of inclusion complexes and thereby are widely used for pharmaceutical applications. In addition, the advantages of CDs as a carrier also include (1) chemical derivatization by site-specific substitution; (2) the tunable cavity sizes; and (3) low toxicity and immunogenicity (Kurkov and Loftsson, 2013; Syukri et al., 2015). The drug molecules can be partially or entirely inserted into the hydrophobic CD cavity under the driving forces of hydrophobic, electrostatic, van der Waals, charge-transfer interactions, etc. New targeting strategies that introduce receptor groups to CDs’ surface further enhance the site-selective delivery capability of CDs (Guo et al., 2020; Peng et al., 2020). For instance, the receptor peptides and lipophilic groups are typically modified to CDs for penetrating the blood-brain barrier to improve drug treatment. Besides these designed derivatives, CDs may also self-aggregate into supramolecular polymers or networks through hydrogen bonds and host-guest interactions or may be linked to form larger structures via covalent bonds to construct optimal architectures with both high delivery efficiency and low cytotoxicity (Hu et al., 2014). These CD-based drug delivery vectors can be dosed by oral, nasal, ocular, rectal, and dermal delivery and have proven to enhance the drug absorption, mask odors, drug release, and drug permeability through the biological barrier (Brewster and Loftsson, 2007; Carrier et al., 2007).

### Antimicrobial Drug Delivery

**FIGURE 3:**
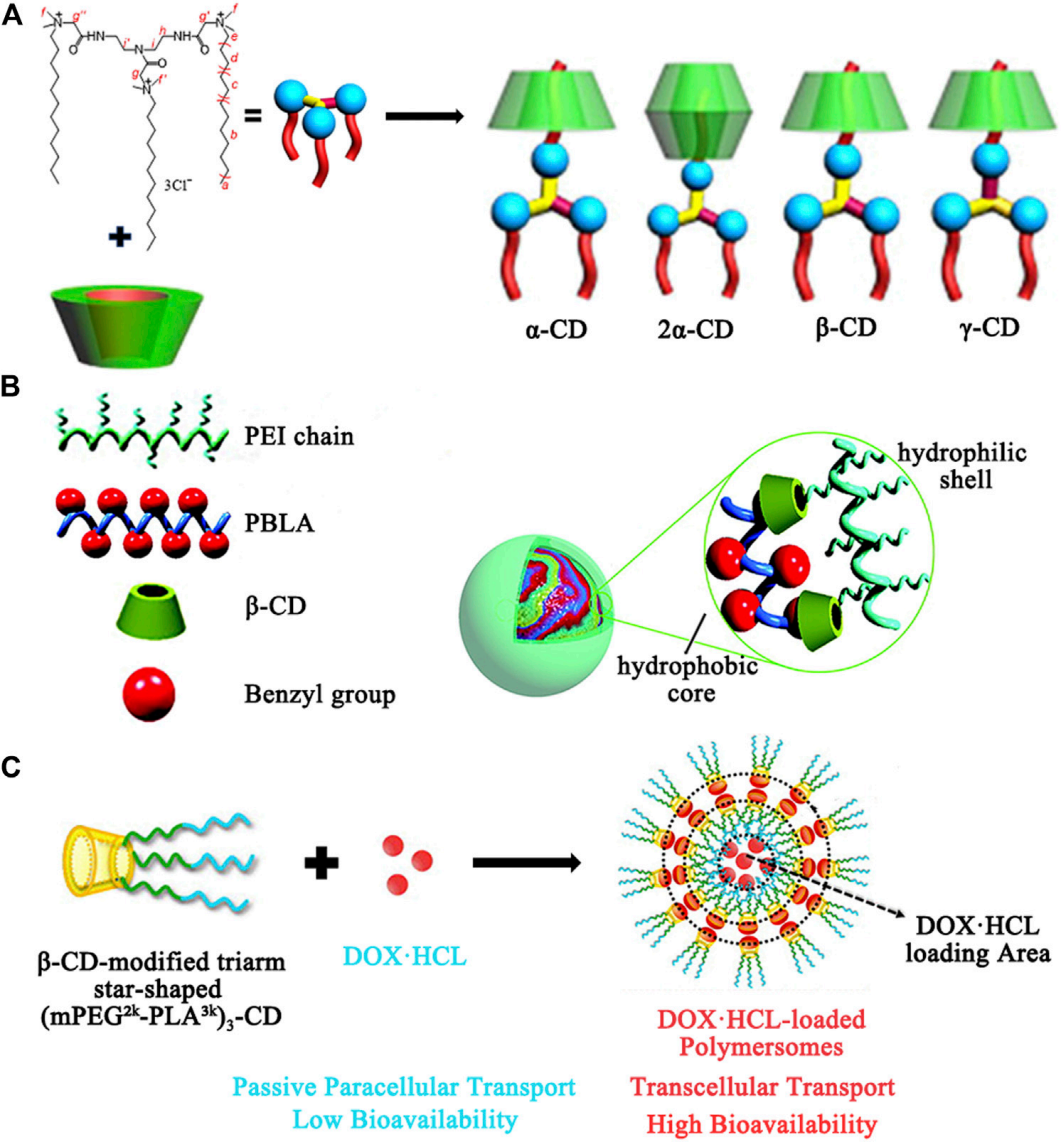
**(A)** Illustration of structure of the complexes of CDs and DTAD. **(B)** Illustration of structure of the core-shell nanoassemblies based on PEI-CD/PBLA by a host-guest interaction for dexamethasone delivery. **(C)** Illustration of the structure and the property of the polymersome for carrying DOX·HCl.

The resistance of bacteria to antibiotics is a serious global problem in the field of medicine. Although new antimicrobial agents such as metal nanoparticles (NPs), quaternized ammonium compounds, carbon nanomaterials, triclosan, herbal extracts, and antimicrobial biopolymers are being developed (Moellering, 2011; Dong et al., 2020), it still hard to neutralize the bacterial defense mechanisms due to the rapid evolution of bacteria. Another effective strategy to overcome antibiotic resistance is to encapsulate antimicrobial agents into a delivery system for the reduced dosages or the targeting characteristics. CDs are the most representative host complexation agents in delivery systems, which can act as reservoirs to improve the undesirable properties of antimicrobial agents. In addition, chemically modified CDs like hydroxypropyl-, methylated-, and sulfobutylether cyclodextrin not only exhibit stronger complexation ability, but also disrupt the crystalline structure of CDs for more efficient drug solubilization. Moreover, some exceptional features (e.g., specific surface area, high charge density, and controlled release ability) can also be obtained by covalently attachment or physically assembly of CDs to a variety of organic/inorganic materials, leading to bacterial cell membrane damage and bacterial death (Cutrone et al., 2017; Liu et al., 2019). As shown in Figure 3A, Wang et al. reported that α-CD, β-CD, and γ-CD formed complexes with a star-shaped cationic trimeric surfactant (DTAD), respectively, which improved the mildness of the DTAD, enhanced interaction with cell membrane, and maintained good antibacterial activity to Gram-negative *E. coli* (minimal inhibitory concentration values are 2.22–2.48 μM) (Zhou et al., 2016a). Maria and coworkers chose β-CD as a stabilizer and glucose as a reducing agent to synthesize silver nanoparticles for antimicrobial (Andrade et al., 2014). In particular, the β-CD stabilizing layer around the silver nanoparticles, which is very related to the nanoparticle stability and biocompatibility, was characterized in detail. The β-CD-coated silver nanoparticles showed a promising bactericidal activity against the microorganism *Escherichia coli* (a minimum inhibitory concentration was 20 *μ*g·ml^−1^).

### Antioxidant Drug Delivery

Reactive oxygen species (ROS), a byproduct of aerobic metabolism, can cause many diseases under excessive conditions in the body (Sies, 1986; Wirth, 2015). The synthesis of artificial antioxidant enzymes (Jiang et al., 2019) and the delivery of natural antioxidant drugs such as flavonoids (Nagula and Wairkar, 2019), usnic acid (Chemico-Biological Interactions 2020, 332, 109297), celastrol (Journal of Molecular Liquids 2020, 318, 113936), and quercetin (Xu et al., 2019) are the two main directions of scavenging excess ROS. In most cases, chemical modification of CDs is still an important method to construct multifunctional antioxidant systems. Site-specific modification of β-CD at 6-OH position with functional substituents like cinnamic acid derivatives leads to significantly enhanced solubility (>600 mg/ml) and new antioxidant function in scavenging DPPH radicals (Colloids and Surfaces A, 2020, 606, 125382). To avoid the drawbacks of these synthetic antioxidative compounds (e.g., nonspecific distribution, rapid metabolism, and low delivery efficiency), a new generation of nanotherapy based on the functionalization of β-CD with an oxidation-labile compound PBAP to form nanoparticles exhibits a broad spectrum of antioxidative properties and is capable of targeting the inflamed gastrointestinal tissues for selective drug release (Biomater. Sci., 2020, 8, 7117). Moreover, CD carriers are excellent delivery systems for encapsulation of antioxidant drugs in order to 1) stabilize these active compounds, 2) prevent the enzymatic/chemical degradation, and 3) improve their solubility, permeability, and pharmacological activity. Liang and coworkers prepared a simple coevaporation method to synthesize the inclusion complex of hydroxypropyl-β-cyclodextrin (HP-β-CD) and phloretin (Wei et al., 2017). The formation of the complex led to a 5808-fold increase in the solubility of phloretin compared with that in pure water at 25°C and still maintained the ability of scavenging DPPH similar to that of free phloretin *in vitro*.

### Anti-inflammatory Drug Delivery

Inflammation is a defensive response of the body to tissue injury. Clinically, the use of anti-inflammatory drugs (e.g., indomethacin, dexamethasone, and ibuprofen) is one of the major routes for the treatment of inflammatory disorders, which may cause some inevitable side effects such as gastric irritation, ulceration, hepatic failure, and skin rashes (Rainsford, 2007). The CDs have the cavities compatible with the polarity, size, and shape of drug structures and are suitable for their encapsulation to extend the physicochemical properties of drug formulations. Furthermore, a combination of CDs and other materials [e.g., polymers, metal-organic framework (Rajkumar et al., 2019), and hydrogels (Arslan et al., 2020)] provides more versatility in nanocarriers engineering, which allows precise and controlled administration of the anti-inflammatory drugs by surface modification of CD nanocarriers for favorable interactions with the inflammatory microenvironment. As shown in Figure 3B, Ma et al. constructed core-shell nanocarriers for delivery of dexamethasone (DMS) and plasmid DNA, simultaneously (Zhang et al., 2010). Polyethyleneimine (PEI) modified with β-CD by a nucleophilic substitution reaction was used as a hydrophilic shell, while poly (β-benzyl  L-aspartate) (PBLA) with benzyl groups forms a hydrophobic core by inclusion interaction between the benzyl group and the CD unit. The hydrophobic core serves as a nanocontainer for DMS, and 5.8% DMS was loaded and the release of DMS from PEI-CD/PBLA assemblies can be sustained for about 1 month. In addition, Chiavacci and coworkers have prepared a metal-organic framework structure with γ-CD as an organic linker (γ-CD-MOF) (Abucafy et al., 2018). γ-CD with symmetric arrangement (C_8_) and good biocompatibility maintain the crystallization of MOF and improve its bioavailability. γ-CD-MOF was proven to effectively capsule and control the release of anti-inflammatory drugs, sodium diclofenac.

### Anticancer Drug Delivery

In recent years, chemotherapeutics and gene therapy have been widely developed for cancer therapy (da Silva-Diz et al., 2018; Liu et al., 2018a). Typically, the inclusion complexes of CDs carriers with some hydrophobic drugs such as doxorubicin, camptothecin, paclitaxel, and fluorouracil can increase their bioavailability and provide better therapeutic effects through membrane absorption enhancing properties and the stabilization ability with drugs (Tian et al., 2020). Besides the limited number of anticancer drug species, CD-grafted polymer transfection systems such as CD-linked PEI and CD inclusion complexes with guest moieties (e.g., adamantane, azobenzene, ferrocene, and cholesterol)-modified poly (ethylene glycol) (PEG) were also developed for antitumor gene therapy by improving gene transfection efficacy (Lai, 2014). Moreover, various responsive nanostructures (e.g., redox-, pH-, Photo-, voltage-, and thermosensitivity) can be built based on sensitive covalent/noncovalent interactions (e.g., disulfide bonds, the interactions between CD and N-methylbenzimidazole, azobenzene, ferrocene, etc.) or polymer properties (e.g., the reversible phase transition of PNIPAA polymers) to achieve controlled release under special tumor microenvironment (Zhang et al., 2020). As an example, β-CD functionalized with hyaluronic acid (HA) and adamantane modified with camptothecin (CPT) are self-assembled into nanocarriers through the specifically host-guest interaction, while a near-infrared absorbing dye IR825 was loaded in hydrophobic cavity (Zhang et al., 2018). In this way, the aqueous solubility of CPT had significantly increased. In tumor microenvironment, disulfide bond breakage leads to CPT release for the chemotherapy. Nevertheless, the dye IR825 could efficiently absorb light energy into heat for the photothermal therapy. Chen et al. (Liu et al., 2018b) used the nucleobase guanine/cytosine (G/C)-terminated PEG and α-CD to prepare supramolecular hydrogels (SHGs), which enhanced DOX-loading efficiency and improved the storage moduli (G’s) of the hydrogels. The SHGs exhibited good biocompatibility and thermal response and were expected to apply in local chemotherapy of cancers. Moreover, as shown in Figure 3C, Qiu and coworkers (Hu et al., 2016) performed pioneering work with developing a DOX·HCl-loaded polymersome (Ps-DOX·HCl) with high drug loading capability to improve oral bioavailability of DOX·HCl, which self-assembled by amphiphilic β-CD-centered triarm star polymer (mPEG^2k^ -PLA^3k^)_3_-CD. In this system, β-CD modification on mPEG-*b*-PLA copolymers can improve the loading of DOX·HCl and change the transmembrane pathway of DOX·HCl. Pharmacokinetic studies in mice showed that the oral bioavailability and extended half-life of the Ps-DOX·HCl were significantly higher than that of free DOX·HCl.

## Molecular Probes for Biomedical Diagnosis

Molecular imaging is an early noninvasive diagnostic method to visualize the physiological or pathological processes at the cellular and tissue level (Herschman, 2003). In general, a typical probe for molecular imaging is composed of imaging element with magnetic, echogenic, radioactive, luminescent, or multimode signals and targeting moiety for specific recognition of the disease-related biomarkers. CDs are considered ideal scaffolds for the design of molecular probes, which have the competitive advantages of (Lai et al., 2017): 1) easy functionalization with targeting ligands and imaging elements to obtain high imaging specificity and multimodal imaging; 2) tunable cavity sizes to optimize their load capacity, imaging time, and excretion behavior; and 3) optical transparency in a broad wavelength range without any signal interferences.

### Probes for Magnetic Resonance Molecular Imaging

Magnetic resonance imaging (MRI) is a rapid and precise diagnostic technique that uses strong magnetic fields and radio waves for high quality imaging of soft tissues. Currently, this method is still confronted with the problems of low magnetic signal and sensitivity, which requires a contrast agent to enhance signal contrast. The construction of paramagnetic contrast agent by modifying CDs with the chelators (e.g diethylenetriaminepentaacetic acid (DTPA) and 1,4,7,10-tetraazacyclododecane-1,4,7,10-tetraacetic acid (DOTA)) is a feasible strategy to improve the relaxation properties and signal intensities by slowing down the rotation of their ion complexes (e.g., Gd3+ complexes). Similarly, the relaxivity value can be modulated by inclusion complexation of CDs or their derivatives with the chelators to restrict the rotational freedom. The targeting capacity is also easily introduced by incorporating specific ligands into CDs for selective recognition of biomarkers. Lu et al. fabricated a CD-based carrier by conjugating β-CD molecules to polyhedral silsesquioxane (POSS) (Zhou et al., 2016b). The adamantane modified cyclic RGDfK peptide and the macrocyclic Gd^3+^ chelate were incorporated into the carrier via host–guest chemistry to generate a targeted contrast agent for cancer. The complex of Gd^3+^ chelate and CD cavity can increase the relaxivity value. The contrast agent leads to strong and prolonged contrast enhancement in tumor tissues, as compared to the nontargeting counterpart in mice bearing 4T1-GFP-Luc2 flank tumors.

### Probes for Ultrasound-Based Molecular Imaging

**FIGURE 4:**
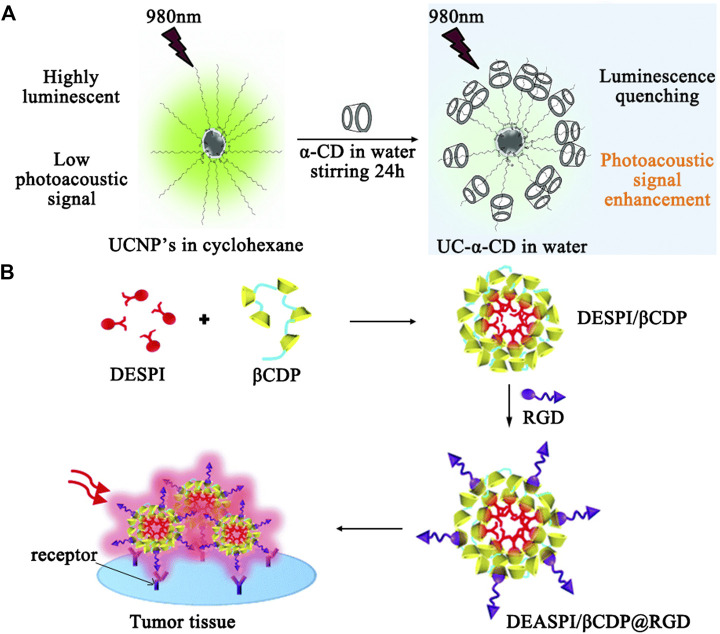
**(A)** Illustration of luminescence quenching effect and subsequent photoacoustic signal enhancement from UC-α-CD in water. **(B)** Illustration of fabrication of the DEASPI/βCDP nanomicelles and application in TPE tumor tissue imaging.

Compared to MRI, ultrasound (US) imaging is a more safe and affordable technique, which evaluates the reflected echoes based on the variations such as sound attenuation effects, backscattering coefficients, and sound speeds. CD-coated microbubbles are new US contrast agents that not only create strong ultrasound reflections by reducing gas dissolution, but also can improve the signal-to-noise ratio of imaging when targeting moieties are incorporated into CDs via host-guest interactions to obtain the enhanced target specificity. As an extension to 2D imaging, ultrasound-based 3D imaging provides more information such as morphological, functional, and molecular data for lesion analysis. Photoacoustic (PAI) imaging is a recently developed hybrid technique that combines the high selectivity of optical imaging and the deep penetration of US imaging using endogenous contrast for real-time molecular imaging. Zhao and coworkers reported an upconversion nanoparticles (UCNP)/α-cyclodextrin (α-CD) inclusion complex (UC-α-CD) for *in vivo* PAI (Maji et al., 2014) (see Figure 4A). UCNP and NaYF_4_ co-doped with ytterbium (Yb^3+^) and erbium (Er^3+^), cannot be dispersed in aqueous solution due to the presence of oleic acid (OA). By forming the inclusion complexes with α-cyclodextrin (α-CD), water-dispersible UCNPs (UC-α-CD) were prepared. Under 980 nm excitation, the luminescence of UC-αCD was quenched to generate enhanced PA signal in aqueous conditions for *in vivo* PAI in live mice. The obtained images of the mouse kidney before and after the injection of UC-α-CD demonstrated the excellent PAI generation capability of UC-α-CD. The surface of UC-α-CD can be further integrated with various functional groups for specific cancer imaging.

### Probes for Radiation-Based Molecular Imaging

Radionuclide imaging is another form of noninvasive imaging technique that uses biologically active compound labelled with a small amount of radioactive substance to detect the disease-associated molecular phenotype of tissues. This technique has significant advantages of high sensitivity low dose, quantitative and qualitative localization, unlimited tissue penetration, and observation of dynamic metabolism, but shows lower spatial resolutioThe selection of different radionuclides, especiallyradioactiveisotopes such as ^99m^Tc, ^111^In and ^131^I that produce γ-rays is crucial for label tracers to produce tomographic images. Based on the early reports, CD are favorable to improve the bioadhesion of radiolabelled probes via hydrogen-bonding interaction. For instance, Peñuelas and coworkers labeled CD-poly (anhydride) nanoparticles (CD-NP) with technetium-99 m (^99m^Tc) for biodistribution studies after oral administration (Areses et al., 2011). Single photon emission computed tomography (SPECT) fused computed tomography (CT) images revealed that CD-NP moved remarkably more slowly inside the gut than conventional NP, derived from stronger interactions between the gut mucosa and CD. Actually, there is still great potential for further optimization of CD-based probes, whose biological half-life is ideally shorter than the half-life of the radionuclide. This allows the non-specific signals to be eliminated within the imaging timeframe, leading to a cleaner signal of interest.

### Probes for Luminescence-Based Molecular Imaging

Luminescence-based imaging is still considered as the most reliable tool to obtainchemical images using fluorescent probe technology. To date, a wide range of fluorochromes/bio-luminescence materials can be used to monitor the real-time behavior of biologically active species (e.g. ROS) by studying their localization or bio-distribution for pathologicalexaminations. The conjugation of CDs to these systems are able to generate selective optical signals for molecular imaging through improving their recognition ability, which has been confirmed by the earlier study using coumarin-methyl--CD to track hydroxyl radicals. Based on the recognition specificity of CDs, various CD-based fluorescent probes (e.g. the bridged bis-CDs-dye complex, metallocyclodextrins, and CD nanomicelles) have been developed for multiplex detection through one-photon or two-photon excitation. Tan and coworkers performed pioneering work with a two-photon absorption (TPA) nanomicelle based on *β*-CD polymer (*β*-CDP) through the inclusion interaction between *β*-CD and *trans*-4-[p-(N,N-diethylamino)styryl]-N-methylpyridinium iodide (DEASPI); then, a cyclic RGD peptide, which selectively recognizes α_v_β_3_ and α_v_β_5_ integrin receptors, conjugated adamantine was anchored on the surface of the *β*-CDP-based nanomicelle to form a TPA bionanoprobe (DEASPI/*β*-CDP@RGD) by the *β*-CD/adamantine host-guest inclusion strategy (Yan et al., 2014) (see Figure 4B). The probe has been successfully applied to the targeted imaging of tumor cells and tissues with bright TPE fluorescence emission penetration depth up to 300 m*μ*.

## Supramolecular Scaffold for Enzymatic Catalysis

Enzymes are highly efficient and selective catalysts that can accelerate reactions by more than 10^17^ folds.(Richard, 2013) The active site is a key structure of the enzyme to bind a particular substrate for suppressing undesired competing reactions. CDs are ideal candidates for the development of enzyme mimics due to their remarkable guest-inclusion capabilities with small hydrophobic compounds. In most cases, the desired functional groups (e.g. the catalytic groups and substrate-binding groups) can be introduced into the core cyclic structure or at the periphery of CDs to create the structural features of natural enzymes for specific substrate binding or activation (Kataky and Morgan, 2003; Dong et al., 2012). Another strategy is to develop CD-based nanomaterials with enzyme-like activity by stabilizing metal clusters/nanoparticles or inducing the formation of larger aggregates, in which the catalytic core possesses multi-metal catalytic sites and thus exhibits strong rate enhancement.(Li et al., 2008) Furthermore, CDs can be also explored as a modulator to control the activity of artificial enzymes based on the reversibility of host-guest interactions. CD-based supramolecular chemistry creates a great diversity in the design of different enzyme models for effective simulation of various catalytic functions..

### Artificial Enzyme

**FIGURE 5:**
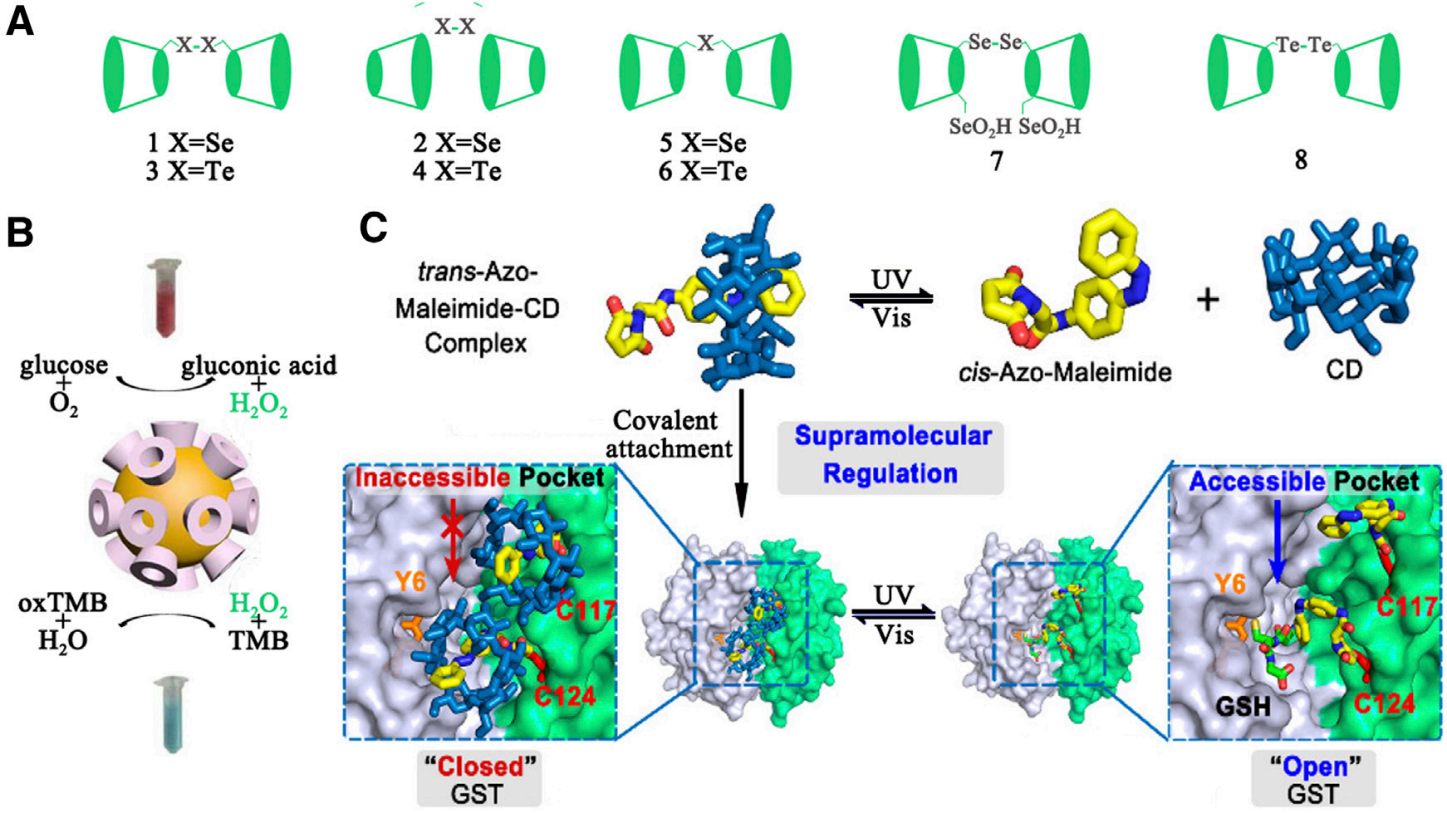
**(A)** CD-based artificial enzymes. **(B)** Illustration of the cascade catalysis system using the -CD@AuNPs as the only catalyst. **(C)** Illustration of the optically controlled supramolecular switch for Glutathione S-Transferase (GST).

As early as 1970s, numerous successful examples have been reported by using natural or modified CDs as supramolecular scaffolds to construct artificial enzymes. In these enzyme designs, site-selective modification at the primary or the secondary hydroxyl groups of CDs was performed to introduce the coenzyme factors, including thiazolium salt, isoalloxazine, phosphate esters, pyridoxamine/pyridoxal, imidazole, oxime anion, metalloporphyrins, and etc. Furthermore, some modifications were also applied to provide additional noncovalent interactions (e.g. electrostatic, hydrogen-bonding, charge transfer, and metal-coordination interactions) or to change the CDs' cavity sizes, shapes, and properties for more favorable geometrical accommodation of the substrates. To date, three different types of CD-based supramolecular enzyme mimics that contain metal free CD-based artificial enzymes, CD-based artificial metalloenzymes, and CD-based artificial enzymes have been created. A variety of enzymatic functions, including oxidase (e.g. CD aldehydes or CD diacids with copper, zinc and iron), reductase (e.g. CD-sandwiched porphyrinatoiron), dehydrogenase (nicotinamide-linked -CD), esterase (tripodal tris(2-aminoethyl)amine (tren)-linked CDhomodimer), aminotransferase (e.g. pyridoxamine-modified -CD), glycosidase (e.g.CD derivatives with cyanohydrin and carboxylate groups), protease (e.g. imidazolyl CD), and nuclease (e.g.copper(II) complex with -CD ) were obtained to rival the activity and specificity of natural ones. In addition, CDs can form a complex supramolecular structure through the complex of host and guest to obtain more excellent enzymatic properties or multiple enzymatic activities. Glutathione peroxidase is a kind of important mammalian selenoenzyme. Liu et al. reported the first cyclodextrin-based artificial enzyme model 1 (Figure 5A) by attaching a diselenide group to the primary face of -CD. Strong substrate binding ability made its GPx activity 4.3 times higher than ebselen. Similarly, enzyme model **2** with a diselenide group bound to the secondary face of -CD was also reported. Due to the stronger binding capacity between **2** and the substrate GSH, **2** exhibited higher catalytic activity. On the basis of **1** and **2**, the same group explored a array of artificial selenoenzymes **3**-**7** based on CDs and investigated their catalytic mechanism. Recently, 6,6'-ditellurobis bridged-CD **(8)** was reported as the best inhibitor which significantly suppressed ferrous sulfate/ascorbateinduced cytotoxicity.

### Nanoenzymes

Some nanomaterials, such as noble metal nanoparticles, ferromagnetic nanoparticles, metal–organic framework, and graphene oxides, have been confirmed to have enzymatic activity. Nanozymes are used to mimic the activity of four types of redox enzymes, including peroxidase, oxidase, catalase, and superoxide dismutase (Wei and Wang, 2013). The unique topology of CDs was often employed to regulate the activity of nanoenzymes (Wang et al., 2018b). For example, Xia and coworkers chose CD molecules as both reducing agents and stabilizers to synthesize gold nanoparticles (CD@AuNPs) for cascade catalysis (Zhao et al., 2016) (see Figure 5A). In the cascade reaction, CD@AuNPs exhibited mimicking properties of both glucose oxidase (GOx) and horseradish peroxidase (HRP) simultaneously and could play a role in the sole catalyst. According to density functional theory (DFT), the authors proposed that the specific topological structures of CD molecules and their unique electron transfer effects with the appended Au surface lead to unpredictable catalytic properties. Furthermore, due to possessing macrocyclic structures on the particle surface, CD@AuNPs can be employed for fluorescent sensing and self-assembly. CDs also could be managed to modify on the surface of nanoenzymes for improving catalytic functions (Vazquez-Gonzalez et al., 2017; Lu et al., 2020).

### Enzyme Switches

The catalytic function of enzymes is usually strictly controlled by various triggering factors during life activities. Typically the “on/off” effect of the variable domain on the catalytic site of an enzyme has a great influence on the enzyme-substrate recognition, and thereby control its catalytic performance reversibly. Inspired by Nature, a large number of artificial intelligence catalysts that contain azobenzene, spiropyran, diarylethylene, and other stimulus response groups have been developed to simulate the strict regulation behavior of natural enzyme (Blanco et al., 2014). CDs with different cavity sizes can be complexed with a variety of stimulus-responsive guest molecules. Luo and coworkers constructed an optically controlled supramolecular switch for Glutathione S-Transferase (GST) (Liu et al., 2017b) (see Figure 5B).The host-guest system of azobenzene/CD was introduced into the catalytic pocket of GST. The photoisomerization of *cis*-azobenzene and *trans*-azobenzene lead to the dissociation and recombination of CD, and the later hinders the binding of the substrate GSH to the active site. The optical-controlled switch is still active after several cycles. Similarly, the same light-controlled switch was also used in palladium-catalyzed bioorthogonal reactions (Wang et al., 2018b).

## Molecular Receptors for Biochemical Sensing

Biosensors for diabetes and pregnancy tests have been commercialized and used in large numbers (Wu et al., 2019b). Moreover, the application of sensors for other biomolecules has also made incredible progress (Luong et al., 2008). However, there are still some challenges in the design of biosensors that can meet actual application requirements, such as designing biosensors with higher specificity, repeatability, sensitivity, and immobilization of biosensors (Jensen, 2006; Pinalli et al., 2018). With the development of supramolecular chemistry, CDs as molecular receptors have been introduced into biosensors (Smith et al., 2015). According to the different properties of analytes, a variety of cavity sizes and functionalized CD derivatives can be employed (Fragoso et al., 2002; Shi et al., 2014).

### Electrochemical Sensors

**FIGURE 6:**
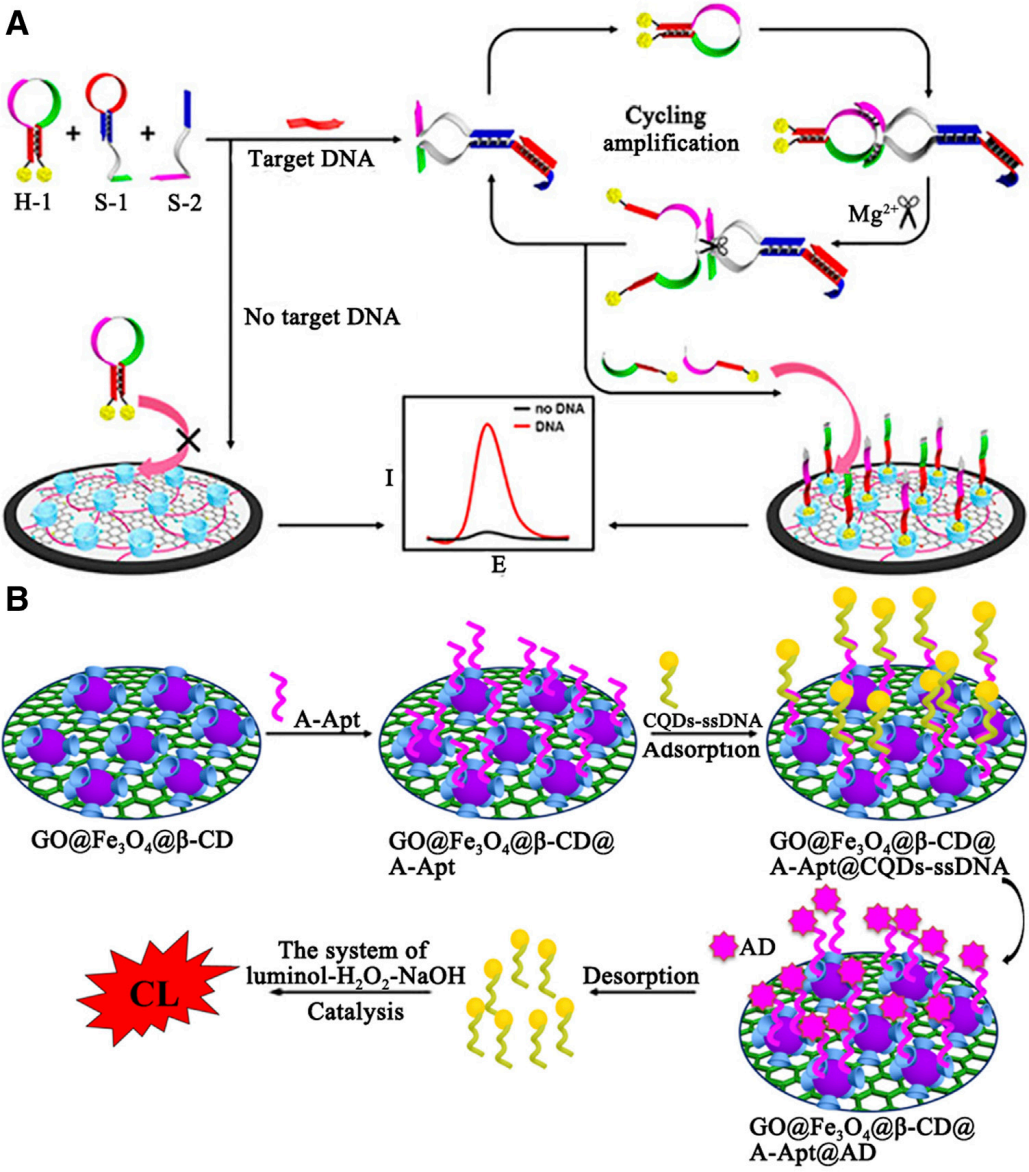
**(A)** Illustration of the electrochemical DNA biosensor based on the host–guest interaction and Mg ^2+^ -assistant DNA recycling. **(B)** The schematic diagram construction of GO@Fe3O4@β-CD@A-Apt/CQDs-ssDNA-CL biosensor.

Electrochemical sensors have received increasing interests, which can be designed simply, ensured reversibly and reproducibly, and analyzed accurately. Diao and coworkers demonstrated an electrochemical sensor based on nanocomposites and Mg^2+^-dependent DNAzyme for sensitive detection of DNA and miRNA (Jiang et al., 2017) (see Figure 6). β-Cyclodextrin polymer (β-CDP) with high water solubility and recognition capability was adsorbed on the nitrogen-doped reduced graphene oxide (NRGO) with superior electrocatalytic activity and placed on the electrode surface to form a sensing platform. In the presence of a target, the hairpin structure of subunit DNA (S-1) was opened to generate activity of catalytic the cleavage of hairpin probe (H-1). Then H-1 binding to S-1 was divided into two single-stranded oligonucleotides with the assistance of cofactor Mg^2+^. Single-stranded oligonucleotides was recognized by β-CDP according to the principle of dimension matching, resulting in an obvious increase in peak current. In contrast, the formation of Mg^2+^-dependent DNAzyme was inhibited, leading to a weak current response. In the sensitive determination of DNA, the calculated detection limit was 3.2 fM, at a signal-to-noise ratio of 3 (S/N = 3). Furthermore, the authors applied this method to the detection of target miRNA. The detection limit was estimated to be 18 fM (S/N = 3).

### Optical Chemical Sensors

Optical chemical sensors based on fluorescence or chemiluminescence (CL) were also employed for biomolecule detection. In Figure 6B, Luo and coworkers designed a highly selective and sensitive CL biosensor for adenosine (AD) detection (Sun et al., 2018). The author first synthesized GO@Fe_3_O_4_@*β*-CD as a sensing platform. Among them, *β*-CD provides a binding site for adenosine polymers (A-Apt), which is a kind of synthetic single-stranded oligonucleotides. Then, CQDs that could be catalyzing the CL system of luminal-H_2_O_2_-NaOH was modified by ssDNA (a single stranded DNA partially complementary to A-Apt). When AD existed, CQDs-ssDNA was released from the surface of GO@Fe_3_O_4_@β-CD@A-Apt and catalyzed CL. The detection limit was 2.1 × 10^–13^ M. Finally, the sensor has been successfully applied to detection of AD in urine samples and recoveries ranged from 98.6 to 101.0%.

## Conclusion

This review summarized the recent development of CD-related systems including CD derivatives and their supramolecular assemblies, which provide versatile platforms and useful properties in the construction of supramolecular materials by forming the inclusion complexes. Some representative examples clearly demonstrate the functional diversity and excellent performances of CDs when having covalently/noncovalently linked bioactive moieties, which endow them with desirable abilities for biomedical applications in bioseparation, enzymatic catalysis, biochemical sensing, biomedical diagnosis, and therapy. Despite the remarkable advances in these fields, the structure-activity relationship is still complicated and remains to be further explored for the improved efficiency. Moreover, new concepts and theories should be conceived to guide the performance-targeted design and construction of more advanced CD-based functional systems.
